# Histone Deacetylase Inhibitor Therapy in Epithelial Ovarian Cancer

**DOI:** 10.1155/2010/458431

**Published:** 2009-12-20

**Authors:** Noriyuki Takai, Hisashi Narahara

**Affiliations:** Department of Obstetrics and Gynecology, Faculty of Medicine, Oita University, Oita 879-5593, Japan

## Abstract

Since epigenetic alterations are believed to be involved in the repression of tumor suppressor genes and promotion of tumorigenesis in ovarian cancers, novel compounds endowed with a histone deacetylase (HDAC) inhibitory activity are an attractive therapeutic approach. In this review, we discuss the biologic and therapeutic effects of HDAC inhibitors (HDACIs) in treating ovarian cancer. HDACIs were able to mediate inhibition of cell growth, cell cycle arrest, apoptosis, and expression of genes related to the malignant phenotype in a variety of ovarian cancer cell lines. Furthermore, HDACIs were able to induce the accumulation of acetylated histones in the chromatin of the p21^WAF1^ gene in human ovarian carcinoma cells. In xenograft models, some of HDACIs have demonstrated antitumor activity with only few side effects. Some clinical trials demonstrate that HDACI drugs provide an important class of new mechanism-based therapeutics for ovarian cancer. In this review, we discuss the biologic and therapeutic effects of HDACIs in treating ovarian cancer, especially focusing on preclinical studies and clinical trials.

## 1. Introduction

Ovarian cancer is the most lethal gynecologic malignancy [1]. Early-stages of ovarian cancer are frequently asymptomatic and difficult to detect and thus diagnosis usually occurs after the disease advanced. The search for agents effective in the treatment of either advanced or recurrent ovarian cancer has been disappointing. To date, platinum and paclitaxel demonstrate the greatest efficacy [1]. However, although reported response rates have been as high as 70%, the duration of response remains brief. In patients with stage III and IV disease, the median duration of response (as measured by progression free survival) following first line therapy is approximately 18 months (reviewed in [2]). Therefore, innovative approaches are needed for the treatment of ovarian cancer.

### 1.1. Histone Modification

One of the most important mechanisms in chromatin remodeling is the posttranslational modification of the N-terminal tails of histones by acetylation, which contributes to a “histone code” determining the activity of target genes [3]. Transcriptionally silent chromatin is composed of nucleosomes in which the histones have low levels of acetylation on the lysine residues of their amino-terminal tails. Acetylation of histone proteins neutralizes the positive charge on lysine residues and disrupts nucleosome structure, allowing unfolding of the associated DNA with subsequent access by transcription factors, resulting in changes in gene expression. Acetylation of core nucleosomal histones is regulated by the opposing activities of histone acetyltransferases (HATs) and histone deacetylases (HDACs). HDACs catalyze the removal of acetyl groups on the amino-terminal lysine residues of core nucleosomal histones, and this activity is generally associated with transcriptional repression. Aberrant recruitment of HDAC activity has been associated with the development of certain human cancers [4]. HDAC inhibitors (HDACIs) can inhibit cancer cell growth in vitro and in vivo, revert oncogene-transformed cell morphology, induce apoptosis, and enhance cell differentiation [5].

### 1.2. Mechanisim of Action of HDACI

HDACs catalyze the removal of acetyl groups from the chromatin core histones. HDACs induce neutralization of the charge on the histones which allows the phosphate backbone of the DNA to open up and therefore facilitate the transcription of many genes, including tumor suppressor genes silenced in cancer. Moreover, acetylation of histones facilitates destabilization of DNA-nucleosome interaction and renders DNA more accessible to transcription factors [6]. In parallel to effects on gene expression and differentiation, HDACIs have also been shown to be efficient inducers of apoptosis in several cellular systems [7]. The precise mechanisms of this effect are under investigation, with suggestions ranging from effects on cellular networks to oxidative stress induction and to DNA damage induction [8].

### 1.3. Different Classes of Drug

Several classes of HDACIs have been identified, including (a) organic hydroxamic acids (e.g., Trichostatin A (TSA) and suberoyl anilide bishydroxamine (SAHA)), (b) short-chain fatty acids (e.g., butyrates and valproic acid (VPA)), (c) benzamides (e.g., MS-275), (d) cyclic tetrapeptides (e.g., trapoxin), and (e) sulfonamide anilides [9] (see Table 1).

### 1.4. Postulated Downstream Effects of Inhibition

**Figure 1:**
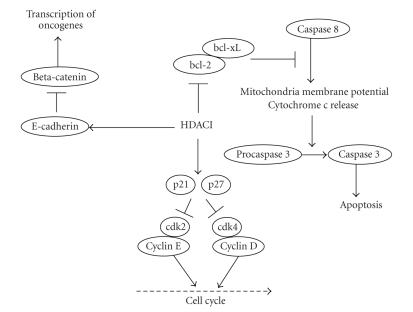
The mechanism of action of HDACIs against ovarian cancer [9].

HDACIs markedly upregulated the level of p21^WAF1^ and p27^KIP1^ proteins, which were expressed at negligible levels in the untreated ovarian cancer cell lines. Conversely, HDACIs decreased the levels of cyclin D1 and cyclin D2. HDACIs decreased bcl-2 levels. E-cadherin binds to *β*-catenin and can act as a tumor suppressor gene; its promoter has CpG islands which are frequently methylated in selected cancers. Although some investigators believed that the expression of E-cadherin can promote carcinogenesis from normal ovarian surface epithelial cells unlike the other carcinomas [10], HDACIs markedly increased the expression level of E-cadherin in endometrial and ovarian cancer cells and exhibit antiproliferative activity in these cells [11] (Figure 1).

## 2. Preclinical In Vitro Studies

SAHA (vorinostat) is one of the most promising HDACIs in treatment of epithelial ovarian cancer. To date, three studies have evaluated vorinostat in ovarian cancer. Takai et al. elucidated for the first time that vorinostat caused cell cycle arrest and markedly induced apoptosis in nine ovarian cancer cell lines [11]. Second, Sonneman et al. found that vorinostat had cytotoxic activities and caspase-3 activities in three ovarian cancer cell lines as well as in primary cancer cells that were isolated from malignant ascites collected from five patients with stage III ovarian carcinomas. They also found that paclitaxel-resistant ovarian cancer cell line (2780AD) cells were responsive to varinostat [12]. Third, Cooper et al. reported that in an ovarian cancer cell line, vorinostat decreased viability and increased apoptosis similarly to paclitaxel, but the combination was not statistically significantly different from the single agents [13].

The anticonvulsant VPA has HDAC inhibitory activity [14]. VPA has an extensive safety history and well-established pharmacokinetics. In cell culture models, exposure to VPA results in dose-dependent cell cycle arrest as well as apoptosis in nine ovarian cancer cell lines [11]. Furthermore, Lin et al. suggested that VPA synergizes with cytotoxic anticancer agents [15].

HDACIs that demonstrated antiovarian cancer activity in single agent are TSA [11], vorinostat [11], CBHA [16], scriptaid [17], sodium butyrate [11], VPA [11], MS-275 [18], M344 [19], apicidin [20], and PDX101 [21].

There are some combination studies in ovarian cancer cells looking at HDACIs in combination with multiple different agents; these include traditional cytotoxic agents (paclitaxel [12, 13, 21, 22], docetaxel [21], cisplatin [15], carboplatin [21]), biologic agents (bortezomib [23]), and aspirin [19]. All of these combination studies in ovarian cancer seek to capitalize on the multiple different mechanisms of action of HDACIs in order to create a synergistic effect with the other modalities and to increase the tumoricidal impact.

## 3. Preclinical In Vivo Studies

We previously tested the ability of VPA to inhibit the growth of human SK-OV-3 ovarian cancer tumors growing in immunodeficient mice during 5 weeks of therapy [11]. Administration of VPA remarkably suppressed the growth of the tumors. During the study, all the mice were weighed once per week. No significant differences in the mean weights, histology of internal organs, mean blood chemistries including liver parameters and hematopoietic values were found between diluent-treated mice and those that received 5 weeks of therapy. It meant that there was no side effect during VPA treatment. Histological analysis of these tumors from untreated mice revealed moderately differentiated carcinomas with small foci of necrosis and fibrosis. Approximately 50%–60% of each of the tumor sections from mice treated with VPA revealed necrosis and histologic changes of apoptosis including formation of apoptotic bodies. These tumors were sampled for expression of p21^WAF1^ using immunohistochemistry on formalin-fixed paraffin-embedded sections. SK-OV-3 ovarian cancer cells treated with VPA showed strong nuclear staining. Control cancer cells from untreated mice had negative or focal weak staining for p21^WAF1^. p21^WAF1^ is cyclin dependent kinase inhibitors (CDKIs) that bind to cyclin-dependent kinase complexes and decrease kinase activity and may act as key regulators of the G0/G1 accumulation (reviewed in [24]).

Qian et al. demonstrated that PXD101 displayed single-agent antitumor activity on human A2780 ovarian cancer xenografts which was enhanced when combined with carboplatin [21]. Cooper et al. reported that a nude mouse ovarian cancer model found limited single agent efficacy with vorinostat; however, paclitaxel followed by vorinostat and paclitaxel alone increased survival compared to either vorinostat alone or vorinostat followed by paclitaxel [13]. These studies raised several questions regarding the optimal sequencing of future combination therapy with HDACIs and chemotherapy.

## 4. Clinical Trials

HDACIs require a significant period of exposure (≥24 hours) to achieve maximum tumor cell killing in culture, presumably because of their action as cell cycle agents. Sequestration and elimination may also be problems in vivo. Thus continuous administration may be required to achieve efficacy in the clinic [9]. Some HDACIs (e.g., TSA and trapoxin) are of limited therapeutic use because of poor bioavailability in vivo as well as toxic side effects at high doses. Sodium butyrate and phenylbutyrate are degraded rapidly after IV administration (short half life) and therefore require high doses exceeding 400 mg/kg/day [25]. Furthermore, these compounds are not specific for HDACs because they also inhibit phosphorylation and methylation of proteins as well as DNA methylation [26].

There is only one phase I data including ovarian cancer patients treated with HDACI. Camacho et al. conducted phase I dose escalation clinical trial of phenylbutyrate sodium administered twice daily to patients with advanced solid tumors at Memorial Sloan-Kettering Cancer Center. Administration of phenylbutyrate sodium in a twice-daily infusion schedule is safe. The maximum tolerated dose is 300 mg/kg/day [27].

The multi-institutional phase II trial assessed the activity and toxicity of a new histone deacetylase inhibitor, vorinostat in patients with recurrent or persistent epithelial ovarian, or primary peritoneal carcinoma [28]. The initial dose of vorinostat was 400 mg orally daily and a cycle was defined as a period of 3 weeks (21 days) and was given at a fixed daily dose until progressive disease or adverse effects prohibited further therapy with this agent. The primary endpoints were progression-free survival (PFS) at 6 months and toxicity. Two women of twenty-seven enrolled patients survived progression-free over 6 months, with one having a partial response. The estimated probability of PFS for at least 6 months was 7.4% (90% C.I. was 1.3%–21.5%). Major grade 4 toxicities were leucopenia and neutropenia (7%). While there has not been clear evidence of QTc prolongation due to vorinostat in either preclinical or clinical studies to date, isolated clinical events of QTc prolongation have been reported for other HDAC inhibitors [29]. This phase II GOG study of vorinostat in recurrent ovarian cancer patients demonstrated that, in this platinum-resistant or refractory patient population, there is limited efficacy for this drug as a single agent. Authors discussed that it could be classified as a biologic response modifier rather than a traditional cytotoxic agent. In ovarian cancer, the potential role for this drug may be in overcoming chemotherapy resistance in recurrent disease or in combination with paclitaxel and platinum agents in the upfront treatment. Due to the nature of vorinostat, it may be more effective in low-volume disease for stabilization or prevention of recurrence. Future preclinical and clinical trials will need to focus on potential synergistic effects of vorinostat with other agents, particularly paclitaxel and platinum agents.

Phase II study, single-arm study of hydralazine and magnesium valproate added to the same schedule of chemotherapy on which patients were progressing, has been conducted [30]. Patients received hydralazine at 182 mg for rapid, or 83 mg for slow, acetylators, and magnesium valproate at 40 mg/kg, beginning a week before chemotherapy. Response and toxicity were evaluated. Seventeen patients were evaluable for toxicity and 15 for response. A clinical benefit was observed in 12 (80%) patients: four PR, and eight SD. The most significant toxicity was hematologic.

There were two clinical presentations from ASCO 2008 with PDX101 (belinostat) both alone and in combination with chemotherapy in ovarian cancer [31, 32]. Mackay et al. demonstrated a phase II trial of belinostat in patients with platinum resistant epithelial ovarian cancer (EOC) and borderline ovarian tumors. Belinostat 1,000 mg/m^2^/day was administered IV on days 1–5 of a 21-day cycle. Tumor response was assessed by RECIST and CA125 criteria every 2 cycles. Of 18 patients with EOC, 9 patients have SD, 6 progressive disease (PD), 3 are nonevaluable (NE), and 2 remain on study. Of 12 patients with borderline tumors, 1 patient had a partial response (PR), 9 SD, and 2 are NE. 1 further patient had a CA125 response. 5 patients remain on study. The most frequent grade 3 adverse events (both patient groups) were bowel obstruction, thrombosis, dyspnea, fatigue, lymphopenia, elevated ALP, and nausea. Belinostat shows promising activity in borderline ovarian tumors. Finkler et al. conducted phase II multicenter trial of belinostat, carboplatin, and paclitaxel in patients with relapsed epithelial ovarian cancer. BelCaP (Bel 1,000 mg/m^2^ × 5 days; carboplatin AUC 5 × 1 day 3; paclitaxel 175 mg/m^2^ × 1 day 3) was given in 3-week cycles. The primary endpoint was overall response rate (OR). OR was 31%, including 1 complete response and 10 PR. In addition, 16 patients (46%) had SD.

## 5. Conclusions

In this review we summarize recent studies on the use of HDACIs especially in human ovarian cancer cells. Many questions are currently still unanswered with respect to HDACI specificities for definite tumor subtypes and the molecular mechanisms underlying HDACI-induced differentiation, cell cycle arrest and apoptosis, and the regulation mechanisms of the specific gene expression and recruitment of HDAC complex to the specific promoter sites remain still to be determined. Also, it is still unclear to what extent different HDACs exhibit different and potentially overlapping functions, and it is important to distinguish the HDAC specificity of HDACIs for the development of selective therapy on the molecular level. Certainly, further work will be required to improve the understanding on why transformed cells are more susceptible to the effect of HDACIs than normal cells. Also, combinations of HDACIs with differentiation-inducing agents, with cytotoxic agents, and even with gene therapy may represent novel therapeutic strategies and new hope on the horizon in the treatment of ovarian cancer.

## Figures and Tables

**Table 1:** Overview of frequently used histone deacetylase inhibitors being available for clinical and research purposes.

Substance groups	Derivatives	Isotype	Study phase
Hydroxamates	Trichostatin A (TSA)	I, II	
	Suberoylanilide hydroxamic acid (SAHA, vorinostat)	I, II, IV	III
	LBH589 (panobinostat)	I, II, IV	II
	PCI24781 (CRA-024781)	I, IIb	I
	LAQ824	I, II	I
	PXD101 (belinostat)	I, II, IV	II
	ITF2357	I, II	II
	SB939	Unknown	I
	JNJ-16241199 (R306465)	I	I
	m-carboxycinnamic acid bishydroxamide (CBHA)		
	Scriptaid		
	Oxamflatin		
	Pyroxamide		
	Cyclic hydroxamic acid containing peptides (CHAPs)		

Short chain fatty acids	Butyrate	I, IIa	II
	Valproate	I, IIa	II
	AN-9		II
	OSU-HDAC42		

Benzamides	MS-275 (entinostat)	1, 2, 3, 9	II
	MGCD0103	1, 2, 3, 11	II
	Pimelic diphenylamide	1, 2, 3	
	M344		
	N-acetyldinaline (CI-994)		II

Cyclic tetrapeptides	Apicidine	I, II	
	Trapoxins		
	HC-toxin		
	Chlamydocin		
	Depsipeptide (FR901228 or FK228) (romidepsin)	1, 2, 4, 6	II

Sulfonamide anilides	N-2-aminophenyl-3-[4-(4-methylbenzenesulfonylamino)-phenyl]-2-propenamide		

Others	Depudecin		
	NDH-51		
	KD5150	Pan-HDACI	

Class I: HDAC 1, 2, 3, 8; class IIa: HDAC 4, 5, 7, 9; class IIb: HDAC 1, 2, 3, 8; class III: HDAC 6, 10; class IV: HDAC 11.
